# THE FEASIBILITY OF A PEER-DELIVERED PSYCHOSOCIAL INTERVENTION FOR FAMILY CARE PARTNERS OF HOSPICE CANCER PATIENTS

**DOI:** 10.1093/geroni/igae098.3140

**Published:** 2024-12-31

**Authors:** Jacquelyn Benson, Kyle Pitzer, Ryan Lindsay, Karla Washington, Debra Parker Oliver

**Affiliations:** Washington University in St. Louis, St Louis, Missouri, United States; Washington University in St. Louis, St Louis, Missouri, United States; Washington University in St. Louis, St Louis, Missouri, United States; Washington University in St. Louis, St Louis, Missouri, United States; Washington University in St. Louis, St Louis, Missouri, United States

## Abstract

Providing care and support to a hospice patient with cancer can exact an enormous psychological toll on families. Approximately 50% of advanced cancer family care partners report moderate to severe depression and anxiety. Many studies indicate that the psychological suffering of family care partners can be alleviated by enhancing their coping skills. However, mechanistically informed psychosocial interventions that are better tailored to the unique needs of cancer caregivers are needed. Study objectives were to: (1) evaluate the feasibility and acceptability of a 1:1 peer-delivered psychosocial support intervention for hospice family care partners of cancer patients, and (2) determine a range of potential effects of the intervention on mechanisms of change and care partners’ psychological distress symptoms to guide variable inclusion for a future trial. The study was carried out using a convergent parallel mixed methods design. Qualitative data were analyzed using deductive thematic analysis. Descriptive statistics were performed for demographic characteristics, and rates of recruitment, attrition, and survey completion. Parametric and non-parametric t-tests were conducted to examine mean differences of study outcomes between baseline and midpoint, and endpoint. Mean differences above 0, in the expected direction, and confidence intervals with a 10% or greater change in the total outcome were used to guide decision-making about future trial inclusion. Results demonstrated that the intervention is acceptable, appropriate, and feasible to deliver to hospice family caregivers of cancer patients. Depressive symptoms, anxiety symptoms, benefit finding, and self-efficacy were also determined to be appropriate measures for future use in a larger trial.

